# Assessment of without prescription antibiotic dispensing at community pharmacies in Hazara Division, Pakistan: A simulated client’s study

**DOI:** 10.1371/journal.pone.0263756

**Published:** 2022-02-17

**Authors:** Tawseef Ahmad, Faiz Ullah Khan, Sayyad Ali, Asad Ur Rahman, Shujaat Ali Khan

**Affiliations:** 1 Department of Pharmacy, COMSATS University Islamabad-Abbottabad Campus, Abbottabad, Pakistan; 2 Department of Pharmacy Administration and Clinical Pharmacy, School of Pharmacy, Xi’an Jiaotong University, Xi’an, China; The University of Jordan School of Pharmacy, JORDAN

## Abstract

Antibiotics dispensing without a prescription is an irrational practice and can increase the risk of antibiotic resistance, which is a significant public health concern around the globe. This study was aimed to determine the extent to which antibiotics are supplied without prescription in the community pharmacies (CPs) at Hazara Division from November 2020 to February 2021. The simulated client method (SCM) was used, and the data were gathered, recorded, and analyzed through different statistical methods with descriptive and inferential techniques. The antibiotic dispensing was observed in CPs (90.5%), the most dispensed antibiotics were azithromycin (29.4%) and ciprofloxacin (46.5%) respectively. Furthermore, visited medical stores/ drug outlets, 9.5% of the visited stores denied dispensing of antibiotics because they preferred a referral to visit physicians (23. 9%) and (12.8%) did not had the antibiotics at the time of visits. Antibiotics were more obtainable in retail medical stores (AOR = 8.6, 95 percent Cl: 3.0–24.7; *p* = 0.001) than in pharmacies. In rural areas antibiotics dispensing was more (p = 0.004) as compared to urban areas. Staff members also had asked about patient’s (17.7%) symptoms and drug allergies (12.3% and 3.9%), and (1.5%) they consoled them about their medications. The findings of this study indicate that nonprescription antibiotic sales are very common, despite national rules prohibiting this activity. When the simulated Client requested for any medication to relieve his or her discomfort, many antibiotics were given out without a prescription. Pharmacies/medical stores in Hazara Division selling antibiotics without a prescription are worrying and need immediate action by regulators.

## Introduction

Globally, antibiotic resistance (ABR) is a major public health threat that demands urgent worldwide mediation [1, 2]. To deal with this issue, 68th world health assembly passed a resolution on WHO recommendations based on the global action plan for antimicrobial resistance (AMR), that directs all member states to establish a national strategic plan for the AMR [3–5]. Among five objectives blueprint, one of the strategic objectives is to optimize the antibiotics use as suggested by global action plan against AMR [1].

The unreasonable, irrational, and indiscriminate consumption of antibiotics, admitting for self-medication of antibiotics which leads to ABR [5]. In the decade of 2000 to 2010, the use of antibiotics is increased by 35% with lower and middle-income countries (LMICs) such as Russia, Brazil, China, India, and South Africa accounting for 76% of the increase [6]. In developing countries, the misuse of antibiotics is deeply worrying. Pakistan, Nepal, Eritrea, Uzbekistan, Oman, Sudan, Jordan, Zimbabwe, Lebanon, Yemen, and Nigeria have been reported with higher rates of antibiotic usage [7]. The inappropriate consumption of antibiotics is not usually in developing countries only, but as well in far-flung in the developed world [8]. Ronning et al has reported a study in 16 European countries that discovered a higher rate of antibiotics than required in these countries [9]. ABR is on rise due to the widespread misuse of antibiotics and which making them less effective [10, 11]. Antibiotic-resistant bacteria cause more than 2.8 million illnesses only in the United States each year, with more than 35,000 people dying as a result. Antibiotic resistance increases direct health-care expenditures by $20 billion [12]. Ye et al accounted that almost 80% of the public in China bought antibiotics without a prescription from community pharmacies for self-medication [13].

Overall, It has been estimated that more than one-half of medications are prescribed, dispensed, or sold inappropriately across the world [14, 15]. Worldwide, approximately 700,000 people die because of drug-resistant infection with the possibility of arising to 10 million by 2050 if no action has been taken [1]. Irrational and inappropriate use of antibiotics is a global problem, specifically in Asian region [16, 17]. Inappropriate use of antibiotics is commonly seen for a self-limited viral infection like as URTIs (Upper respiratory tract infections) [18], acute diarrhea [19], and also for some bacterial infections including UTIs (Urinary tract infections) [20] the great contributing factor to inappropriate consumption of antibiotics is self-medication in the community [21].

In Pakistan, 96% of community pharmacies /medical stores dispensed antibiotics without prescription [5]. Easy availability of antibiotics is a major cause of resistance with pressure on physicians to prescribe and a pharmacist to dispense antibiotics for predominantly self-limited conditions, in lower-middle-income countries (LMICs) assisted by inadequate hygienic and living conditions, firing infection rates [17, 22]. In some countries, antibiotics are highly dispensed without a prescription in community pharmacies adding to antimicrobial resistance (AMR) rates, particularly if antibiotics are dispensed for infections caused by a virus such as cold and cough [23–25]. WHO conducted a survey in China, Sudan, and Egypt about 56% of the population ended taking antibiotics as they began to feel improved [3]. To sustain the loyalty of clients and to forbid losing them to a neighbor’s competitor, community pharmacists are dispensing antimicrobials without a prescription [25, 26]. We are cognizant of an extravagant rate of self-medication of antimicrobials and other medications in Pakistan frequently for economic reasons [13, 27, 28]. Despite the Drug Act 1967 amended in 1976 of Pakistan saying that antibiotics aren’t OTC (over the counter) medicines and their accessibility is illegal without a prescription to the public [29]. Overall 65% increase in antibiotics intake in 2000–2015 [30]. A study from Pakistan describes the inappropriate intake of antibiotics as contributing to the resistance problem across the country [30]. Pakistan established a plan known as the "Antimicrobial National Action Plan," Pakistan upon WHO recommendation with the aim of Using a "One Health" approach associated with the WHO Global Action Plan on AMR, establish a functioning, coordinated, collaborative, and long-term AMR containment system to minimize the AMR in future [31]. Community pharmacists are the 1^st^ healthcare, professionals that patients look up to specifically in LMICs, they are the main neutral with bringing down non-prescription and inappropriate dispensing of antibiotics [32, 33]. Antibiotics dispensing without prescription is one of the major problems in Pakistan and thus the present study was carried out to determine the extent of antibiotics dispensing without prescription in Urban and Rural areas of Hazara Division KPK, Pakistan utilizing the SC methodology.

## Materials and methods

### Study design and setting

A multicenter cross-sectional study was conducted between November 2020 to February 2021 employing the SCM in all districts of Hazara Division, Khyber Pakhtunkhwa, Pakistan. Overall, 1500 drug retail outlets including pharmacies are located in all eight districts and the study sample was drawn from the given population.

Hazara division is a region in Pakistan’s Khyber Pakhtunkhwa province’s north-eastern corner. It lies east of the Indus River and is made up of eight districts: Abbottabad, Battagram, Haripur, Mansehra, Upper Kohistan, Lower Kohistan, Kolai Palas Kohistan, and Torghar. The eight different districts of the Hazara division were selected in this study to cover the diverse characteristics of geographical location (the Extreme North and Urban North) socioeconomic status (High and Low) the density of community pharmacies and drug outlets, and the prevalence of self-medication.

### Study population

In Pakistan, the pharmacies are presently operated by a registered pharmacist, although medical stores are either operated by a pharmacy technician or pharmacist. This study includes 310 community pharmacies and medical stores which provided access to the community throughout the study period. Pharmacy staff (dispensers) was the target as a study through population SCM according to their qualification and presence at the drug sale point.

### Data collection tool and approach

In Pakistan, the pharmacies are presently operated by a registered pharmacist, although medical stores are either operated by a pharmacy technician or pharmacist. There is a total of 1500 pharmacies/drug outlets in the Hazara division as a whole and run by pharmacists, pharmacy technicians, and dispensers, the given numbers were confirmed from the database of the licensing office of health authorities in each district of the division. Using a non-probability convenience sampling technique, a total of 310 pharmacies and drug outlets were included in the study. The sample size was computed with a 5% margin of error (95% CI), and a 50% response distribution through the online sample calculator Raosoft.

For the data collection, a data collection team was developed as per SCM criteria. Total 11 (make 3 groups (per group n = 3) members and 2 members supervised the data collection process) pharmacy graduate students aged between 25–31 years and enrolled in post-graduation from the department of Pharmacy COMSATS University, Islamabad, Abbottabad campus were recruited as simulated clients (pseudo patients). Different pieces of training sessions were conducted, and SC underwent training as practice as an SC as per SCM protocols. Each simulated client was assigned to present the one of pre-defined clinical scenarios (**S1 File: Appendix 1**) for upper respiratory tract infections and urinary tract infections (URTI and UTI). They were paired up to visit the community pharmacies and drug outlets that had been studied, one was assign a job to notice and observe the license, registration, availability of a qualified person at CP, and behavior of the dispenser. The second SC was to observe the dispensed drug name, dose, duration, dosage form, and drug counseling-related information. The selected SC were given all the related important forms including information of the study ahead of time to ensure a seamless performance throughout their visit.

**Fig 1:**
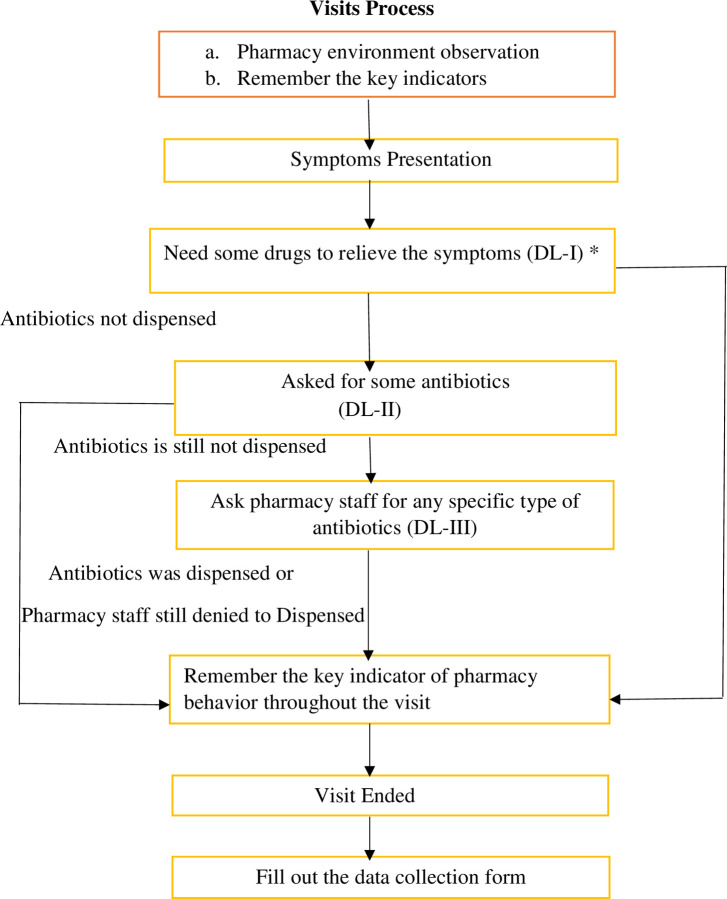
Data collection process: Key indicators involve name, professional license, the appearance of the dispenser, key indicators for dispensing, dispensed antibiotics name and dose, demand level and any alternative medication offers. *DL = Demand Level.

A clinical scenario was designed and presented as to adult URTI and UTI as a pseudo patient/SC, because both infectious conditions are the most frequent illness symptoms linked to antibiotic overuse in both home self-medication and primary health care settings in Pakistan [16, 30, 34]. These two are the most used reported scenarios in previous simulated type studies with great outcomes [23, 35]. The visiting process, symptoms, and preset responses to queries likely to be asked by pharmacy personnel have previously been defined. The visit process and the transcript of the presentation were tested and modified through a pilot survey and pilot results were not included in the final analysis. Investigators addressed the employees as though they were regular clients, avoiding any suspicion. The investigator applied using three levels of DPR (direct product request) till the antibiotics were dispensed or refused (**Fig 1**). Our SC’s initial task was to keep an eye on the pharmacy/medicine counter environment and make a mental note of the key indicators. Then the SC asked like *“I want medications (antibiotics) to relive my symptoms” without presenting a prescription (*request level 1), *“could you please give me antibiotics*?*”* (Demand level 2), and *“I would like amoxicillin’s or macrolides”* (Demand level 3). This approach enabled us to access inappropriate antibiotic dispensing arising from the supply side (Level 1) versus the demand side (level 2 and 3). Macrolides (azithromycin and erythromycin) and amoxicillin were requested because they are most common and used (in clinical settings) among the general public too, hence unlikely to cause any doubt to the pharmacy staff. The SCs were encouraged to memorize each important indicator of pharmacy practice during the visit and then terminated the visit and completed the data collection form at each demand level when antibiotics were provided or refused to dispense.

The SCs reported everything in each new data collecting form **(S2 File: Appendix 02)**, including whether antibiotics were provided or rejected by pharmacy personnel, any advice offered to the simulated client, and anything else. To improve the accuracy of the data collection, the simulated client filled these forms promptly after each visit.

### Statistical analysis

The data were analyzed using statistical software (SPSS) version21. The descriptive and inferential statistic methods were employed, and descriptive analysis was performed on continuous variables with the Standard Deviation and mean. Percentages and frequencies were used to summarize the categorical data. Using bivariate and multivariate logistic regression, the relation between the dispensing of antibiotics without a prescription and variables (type and location of the drug retail outlet, age, and occupation of the dispenser) was investigated. The multivariate analysis includes variables that were found to be significant at the bivariate level.

### Ethical consideration

The ethical clearance was obtained (**S3 File: Appendix 3**) from the ethical committee of COMSATS University, Department of pharmacy, Pakistan (PHM. Eth/CS-M01-022-1703). To protect the anonymity of the data, all ethical and professional concerns were used throughout the SCM study. The names of drug retailers and dealer IDs (identifications) are kept confidential. All findings at the individual level remained secrets, and only aggregate research information to be released.

## Results

The simulated client went to a total of 310 pharmacies/drug stores. Chain community pharmacies, community pharmacies, and medical stores were among the pharmacies/drug outlets visited by the simulated client. All the pharmacies/Drug outlets were run by males. The study population consists of 11.6% pharmacists, 45.8% pharmacy technicians, and others (**Table 1**). Most of the dispensers were aged between 24–28 and 29–34 was 42.9% and 33.5% respectively.

**Table 1:** Sociodemographic characteristics of the dispenser.

Variable		n (%) Frequency
**Title of Dispenser**		
	Pharmacist	36 (11.6)
	Pharmacy Technician	142 (45.8)
	Nursing Degree/Diploma	11 (3.6)
	Health Assistant	121 (39.0)
**Age** (years)		
	24–28	133 (42.9)
	29–34	104 (33.5)
	35–40	63 (20.3)
	>40	10 (3.3)

**Fig 2:**
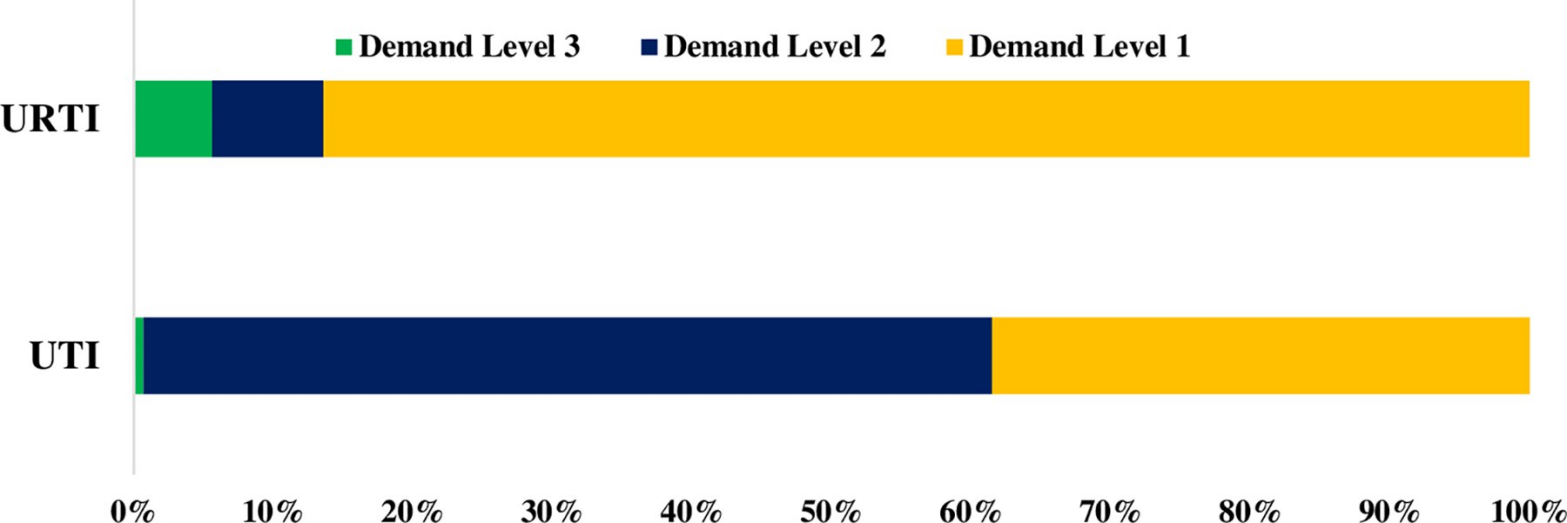
Sale of antibiotics under different level of demands.

Antibiotics were supplied without a prescription to 90.5% SCs, with antibiotics being dispensed in 92.9% and 88.1% of cases, respectively for URTI and UTI. In demand levels 1, 2, and 3, the proportion of antibiotics dispensed without a prescription was 80.3%, 7.4%, and 5.2% respectively (**Fig 2**). Azithromycin was the most prescribed antibiotic (29.4%) and ciprofloxacin (46.5%) (**Table 2**). For separate cases, azithromycin was dispensed by 91 pharmacies in respiratory tract infection and ciprofloxacin was dispensed by 144 pharmacies/drug outlets.

**Table 2:** Antibiotics dispensed without a prescription.

Antibiotics dispensed in RTIs*		Antibiotics dispensed in UTIs*	
Name of Antibiotics	**N (%)**	Name of Antibiotics	**N (%)**
Azithromycin	91 (29.4)	Azithromycin	7 (2.3)
Erythromycin	48 (15.5)	Ciprofloxacin	144 (46.5)
Clarithromycin	15 (4.8)	Gemifloxacin	6 (1.9)
Cephradine	5 (1.6)	Norfloxacin	5 (1.6)
Cefixime	25 (8.1)	Cefixime	53 (17.1)
Moxifloxacin	4 (1.3)	Moxifloxacin	28 (9.0)
Doxycycline	13 (4.2)	Ofloxacin	3 (1.0)
Co-amoxiclav	61 (19.7)	C0-amoxiclav	4 (1.3)
Levofloxacin	26 (8.4)	Levofloxacin	21 (6.8)
**Total**	288 (92.9)	**Total**	273 (88.1)

RTIs: Respiratory Tract infection; UTIs: Urinary Tract Infection.

In the case of non-prescription antibiotics, 9.5% of pharmacies declined to dispense antibiotics without prescription. Sale of Antibiotics Under Different Level of Demands. In 63.3% of the SCs visits, pharmacy staff chooses to refer the patient for additional clinical investigations and denied dispensing antibiotics. The second considerable reasons for refusing antibiotics dispensing were administrative restriction (as by rules and regulations set by local legislation) that antibiotics are not over-the-counter products (23.9%) and inaccessibility of antibiotics at the time of visit (12.8%). Though other than antibiotics medicines like paracetamol along with antiallergic or cough syrups were provided to few clients and in case of UTI, cranberry extract with Syrup for urine alkalinity was given to clients. Strength and doses of dispensed antibiotics for UTI and RTIs are presented in **Table 5** in S1 Table.

The Chi-Square test was used to observe the relationship among antibiotics dispensed without a prescription and independent categorical parameters such as the dispenser’s occupation, type of pharmacy (independent pharmacy, chain pharmacy, and medical store/drug outlet). Local location of pharmacy (urban and rural). Among the tested variables, exact location of pharmacy (*p* = <0.001) and type of pharmacy (*p* = <0.001) were realized to be significantly associated with antibiotics dispensing without prescription.

To determine the degree of the impact and evaluate the continuous variable, a binary logistic regression was used. The age of the dispenser (p<0.08), the type of pharmacy (p<0.0001), and regional location (p<0.004) are seen to be significant and impacting the dispensing of antibiotics in the Bivariate analysis. Medical stores had 14.5 times greater dispensing rates than pharmacies, according to our findings. Rural regions of the division had an 18.6 times greater risk of antibiotic dispensing than urban areas of division. Multivariate regression was used for the same variables as binary regression to adapt the confounding factors. The findings demonstrate that the importance of the type of pharmacy has remained constant. When compared to community pharmacies and chain community pharmacies, medical retailers (AOR = 8.6, 95 percent Cl: (3.0–24.7) were more likely to provide antibiotics without a prescription (AOR = 8.6, 95 percent Cl: (3.0–24.7). (**Table 3**). Questions and advice from the pharmacy staff to the SC patient at the pharmacies in which the antibiotic was dispensed are presented in **Table 4**.

**Table 3:** Bivariate and multivariate analysis with independent and antibiotic dispensing without prescription.

Bivariate Analysis			Multivariate Analysis		
Variables	COR (95% CI)	P-value	AOR (95% CI)		p-value
**Age**					
≤30	Reference		Reference		
>30	0.4 (0.2–1.09)	0.08	0.4 (0.19–1.27		0.14
**Type of pharmacy**					
Community pharmacy	Reference		Reference		
Medical store/ retail outlet (n = 217)	14.5 (5.3–39.7)	<0.001	8.6 (3.0–24.7)		<0.0001
**Location of the pharmacy**					
Urban	Reference		Reference		
Rural (n = 118)	18.6 (2.5–138.9)	0.004	7.1 (0.88–57.0)	0.065	

**Table 4:** Questions and advice from the pharmacy staff to the simulated patient at the pharmacies in which the antibiotic was dispensed.

**Pharmacy Staff Statement**	**Yes**	**No**
Asked few relevant question	55(17.7)	251(81.0%)
Had used other drugs	49(15.8%)	261(84.2%)
Asked more about Symptoms	38(12.3%)	272(87.7%)
Asked about Drug Allergy history	13(4.2%)	295(95.2%)
Counselling or advice on medication	40(12.9%)	270 (87.1%)

## Discussion

To the best of our knowledge, this is the first-ever SCM research study conducted in Hazara division Pakistan to determine the range of sales of antibiotics without prescription. The findings of this study reveal that antibiotics are readily available, which leads to a rise in antibiotic resistance [36]. These findings are analogous to the earlier studies, in which antibiotics are routinely supplied without a prescription at retail shops and pharmacies for the treatment of infections caused mostly by bacteria [37, 38].

In Lahore, Pakistan, multi-center cross-sectional research found that 95% of antibiotics were administered without a physician prescription [5]. Another study performed at five pharmacies in Islamabad and Rawalpindi revealed that over 35% of antibiotics were delivered without a physician’s prescription [39]. Antibiotic dispensing in different regions of the world has been the subject of several research studies [40]. Over-the-counter antibiotic sales predominated globally, according to comprehensive analyses of 35 community surveys from five continents, accounting for 19–100% beyond North America and northern Europe. In terms of our outcomes, Morgan et al. believed that administering antibiotics without a prescription was commonplace for both bacterial and non-bacterial illnesses. More than a third of drug outlets/pharmacies were not asked about medication allergies in 2 studies out of 35, which is quite the same as our study findings [27].

In a study published in Gipuzkoa, a Spanish province (17.5%) [41], Republic of Srpska (18.5%) [42], north-western Spain, antibiotics dispensed without prescription were less likely to be (18.83%) [43], Beirut and its greenbelts, Lebanon (32%) [44], Sri Lanka (41%) [39], Catalonia, Spain (45.2%) [39], and Chinese cities (66.8% and 70%) [45]. The results of a survey performed in Albania (80%) and simulated research conducted in Eritrea (87.6%) were largely consistent with the findings of the previous study [46, 47], while greater results were seen in studies conducted in Moshi municipality, Tanzania (92.3%) Mizan-Aman town, southwest Ethiopia [48]. In the southwest, Ethiopia’s Mizan-Aman town (94.4%) [49], and Zambia’s Lusaka area (100%) [50], Differences in sample sizes, analytical techniques, and simulated instances might explain the discrepancy in the extent found. These data, along with our research, reveal widespread antibiotic abuse in community pharmacies and medical stores across the world, particularly in Pakistan.

Pharmacy personnel dispensed the most azithromycin, ciprofloxacin, and moxifloxacin antibiotics out of the total dispensed. According to research published in Pakistan, azithromycin is the single oral choice for treating extensively drug-resistant (XDR) Salmonella Typhi isolates in Pakistan [51]. According to another study from Pakistan, the most prevalent clinical diagnosis in Pakistan is UTI (Urinary Tract Infection). E. coli was shown to exhibit significant resistance to first-line antibiotics in 28 (30.11%) of the investigations [52]. Azithromycin and ciprofloxacin were the most often supplied and sold antibiotics in our survey, which is concerning. There were also concerns that 15.2% of patients were asked about other medications they were taking, 3.9% of patients were asked about drug allergies, and just 1.5% of pharmacy personnel consoled their antibiotic-using patients. When a major patient is put in danger due to a lack of prescription dispensing, the pharmacy staff’s ability to investigate the associated medicines is tested (Table 3) [42].

The major reasons for antibiotics dispensing without a prescription may include insufficient controls over antibiotics sales, laws enforcement and policies on antibiotics dispensing, and a lack of pharmacists in community pharmacies, according to qualitative research. One antibiotic was dispensed mostly at the demand level, reflecting the current study’s dispensers’ tolerance for the selling of antibiotics and other medications without a prescription. A similar scenario was reported and observed in Ethiopian research, in which antibiotics were mainly administered when an SC sought any drug to ease his or her symptoms [42, 43].

In Pakistan, due to inadequate antibiotic dispensing, pharmacists must play a key role in ensuring the judicious use of antibiotics [53], to reduce AMR rates in the future, based on the national action plan [53]. Pharmacists must provide all important medication administration guidelines and be vigilant in medicine dispensing and AMR in society, based on WHO and FIP(International Pharmaceutical Federation) efforts [54]. Pharmacists should be aware of their responsibilities in addressing patients’ present illnesses, as they are the most accessible healthcare workers to patients in LMICs [55].

In this study, community pharmacists were shown to be less motivated to dispensed antibiotics without a prescription. This study also found that medical shops have a 14.5 times higher chance of dispensing antibiotics without a prescription than community pharmacies. The geographical context of community pharmacy influenced the variance in antibiotics dispensing without prescriptions, which was 18.6 times higher in rural regions of the Hazara division compared to urban areas. This is due to a shortage of pharmacists in rural regions and a lack of knowledge among residents. This study also found a difference in dispensing behaviors, suggesting that the discrepancy might be due to unknown parameters.

Although there was a significant level of engagement by illegal dispensers and non-healthcare professionals. Ciprofloxacin (38.9%) was the most commonly dispensed antibiotic in the case of UTI, similar to Ethiopian [42] and Sri Lanka [43] findings. Ciprofloxacin cross-resistance to other members of the fluoroquinolone family is difficult, especially for those used in second-line anti-TB therapy [56], according to the WHO [44]. Furthermore, pharmacists must be educated in the correct management of infections, which are often seen in ambulatory care services in many states, including Pakistan [28, 34]. In the case of a suspected viral illness such as URTIs, skilled pharmacists administered just a little dose of antibiotics, according to findings [42, 43]. Patients must be encouraged to see their doctors if they are ill. We’ve seen proposals to restrict nations from self-procuring antibiotics come to realization, but the overall impact has been variable. To combat the self-purchasing of medications, multimodal methods, such as penalties and other channels, appear to be the most successful [40].

Meanwhile, the nationwide ban on self-purchasing antibiotics in Pakistan is a source of worry for a variety of reasons. Patients in Pakistan sometimes cannot afford to pay for a physician or medications; therefore, they frequently go to a pharmacy or a drug store for minor diseases. Furthermore, in rural regions, pharmacies/drug stores are sometimes the only healthcare institution with access to drugs. Another factor is the current lack of enforcement of rules in Pakistan. As a result, educating pharmacists and non-pharmacist dispensers to restrict the self-procurement of antibiotics on the WHO’s watch list might be extremely beneficial. In other LMICs, educated pharmacists worked hard to reduce the self-purchasing of antibiotics containing the Republic of Srpska and Kenya following Srpska laws [46].

### Limitation of study

The study population and clinical scenarios chosen may have an impact on the number of antibiotics dispensing simulating other cases such as diarrhea and vomiting. Samples were only taken from the districts’ major towns, which had a better demographic and socioeconomic profile, and only some samples were obtained from the districts’ periphery. As a result, our findings may not be generalizable throughout the country. Regardless, we believe our findings are valid and give direction for the future.

## Conclusion

Despite the regulations, we concluded that giving antibiotics without a prescription is still a prevalent practice in Hazara Division. In addition, the response to simulated clients (pharmacy personnel) did not enquire about the patient’s drug allergies, prior prescriptions, or seek pharmaceutical recommendations. Antibiotic distribution without a prescription is a complicated problem in the community. To address this issue and strengthen policies to guarantee the judicious use of medications, a robust and multi-faceted strategy is required. This research may be followed up by a qualitative study to learn more about what motivates the sale of antibiotics without a prescription.

## Supporting information

S1 Data (RAR)

S1 File Predesigned clinical scenarios. The clinical scenarios was designed for SC to obtained specific antibiotics for these two selected scenarios without prescription.
(DOCX)

S2 File Data collection form. The tool used for the data collection.
(DOCX)

S3 File Ethical certificate. The approval was taken from Department of Pharmacy COMSATS University Islamabad-Abbottabad Campus.
(DOCX)

S1 Table Strength and dose of dispensed antibiotics for the predesigned clinical scenarios (URTI and UTI). Community pharmacist and dispenser dispensed different dose and strength of antibiotics, the most and least both are shown in Table 5.
(DOCX)
