# Supplementary material for: Assessment of without prescription antibiotic dispensing at community pharmacies in Hazara Division, Pakistan: A simulated client’s study
Source: PLoS One. 2022 Feb 17;17(2):e0263756. doi: 10.1371/journal.pone.0263756 (PMC8853528; doi:10.1371/journal.pone.0263756)
Supplement: S1 File — The clinical scenarios was designed for SC to obtained specific antibiotics for these two selected scenarios without prescription. (DOCX) [file pone.0263756.s002.docx]

**Supplementary File: Appendix 01**

**Clinical Scenarios**

**Pre-Designed Clinical Scenario**

| **Clinical case of adult acute respiratory infections,**  A simulated client showing that he has an acute respiratory infection. A 26-year-old Simulated client gives a presentation of his symptoms that he has a fever for the past three days, muscle pain, and a runny nose. Could you provide me some medication to relieve my symptoms?  If asked for more symptoms, the SC will answer, a fever from two days, cough, and headache too.  In response to drug allergy history, the SC answer “No”.  In response to medical history, the SC answer “NO”.  In response to a question if pharmacy staff asked about visiting the doctor? the SC answers "Hasn't".  In response to the prescription, The SC says, "I don't have any prescription".  In case of any referral recommendation, the SC says "it's just a minor illness. I think not required to visit a doctor. Could you just give me advice about medication? |
| --- |

Two clinical scenarios' (URTI's and UTI's) were designed to assess the antibiotics dispensing without prescription in the local community with the possible questions from the pharmacy staff to be asked from the simulated client/patient.

### Clinical Scenario Ⅱ

| **Clinical case of adult Urinary Tract infections,**  A simulated client visits to pharmacy/drug outlet to buy medicines for his elder sister A 32-year-old woman suffering from flank pain, fever, frequent urination for the last 2 days. Could you give me some medicine to relieve her symptoms?  If asked more about pregnancy, the SC will answer "NO".  If asked about other medication use, the SC will answer "NO".  In response to drug allergy history, the SC answer “No”.  In response to medical history, the SC answer “NO”.  In response to a question if pharmacy staff asked about visiting the doctor? the SC answers "Hasn't".  In response to the prescription, The SC says, "I don't have any prescription".  In case of any referral recommendation, the SC says "it occurs frequently to her. I think not required to visit a doctor. |
| --- |
