# Supplementary material for: Assessment of without prescription antibiotic dispensing at community pharmacies in Hazara Division, Pakistan: A simulated client’s study
Source: PLoS One. 2022 Feb 17;17(2):e0263756. doi: 10.1371/journal.pone.0263756 (PMC8853528; doi:10.1371/journal.pone.0263756)
Supplement: S2 File — The tool used for the data collection. (DOCX) [file pone.0263756.s003.docx]

**Supplementary File: Appendix 02**

**DATA COLLECTION FORM**

Number ( ) Scenario ( )TIME: Morning Noon Afternoon

|  | **Section A: Pharmacy Demographics** | | | | | | |
| --- | --- | --- | --- | --- | --- | --- | --- |
| 1 | Pharmacy Name:___________________________________________ | | | | | | |
| 2 | Pharmacy Type | Chain Pharmacy | Independent Pharmacy | | | | |
| 3 | Pharmacy Location | Urban | Rural | | | | |
|  |  | High Economic Level Area | Lower Economic area | | | | |
|  |  | Community | Shopping Mall | Medical Centre | | Other__________ | |
| 4 | Pharmacist Availability | Yes | No | | | | |
|  | **Section B: Information regarding Pharmacy Staff** | | | | | | |
| 5 | Gender | Male | Female | | | | |
|  | Age(Year) | < 30 | 30-50 | | >50 | | |
| 6 | Is qualified person (Pharmacist) On duty? | Yes | No | |  | | |
|  | **Section C: Drug Information Related to Antibiotic Dispensing** | | | | | | |
| 8 | Any advance inquiry about patient’s conditions | Yes | No | |  | | |
| 9 | Antibiotics dispense without prescription | Yes | No | |  | | |
| 10 | Below which demand lever were the antibiotic dispensed without a prescription? | 1. Dispense antibiotics under first demand level i.e., level 1 2. Dispensed under second demand level 3. Dispensed under third demand level | | | | | |
| 11 | Generic Name( Brand) of dispensed antibiotic | ________________Dose___________Duration_____Dosage Form____  ________________Dose___________Duration_____Dosage Form____  ________________Dose___________Duration_____Dosage Form____ | | | Ask reason if not dispensed | | |
|  |  |  |  |  | 1. Need Prescription to dispensed 2. Visit to physician 3. Be caution in using antibiotic 4. Other reason______ | | |
| 12 | Asked few more or less relevant questions | 12.1 Asked for weather had used another drug  12.2 Asked for any other symptoms  12.3 Asked question regarding patient drug allergy | | | Yes  Yes  Yes | | No  No  No |
| 13 | Any referral recommendation | Yes | No | | | | |
| 14 | Counseling or advice provided on dispensed medication | Yes________________ Please record | | | No | | |
| 15 | Alternative medicines dispensed in place of antibiotic | ________________Dose___________Duration_____Dosage Form___  ________________Dose___________Duration_____Dosage Form___  ________________Dose___________Duration_______Dosage Form___ | | | No | | |
