# Supplementary figures and images for: Assessment of without prescription antibiotic dispensing at community pharmacies in Hazara Division, Pakistan: A simulated client’s study
Source: PLoS One. 2022 Feb 17;17(2):e0263756. doi: 10.1371/journal.pone.0263756 (PMC8853528; doi:10.1371/journal.pone.0263756)

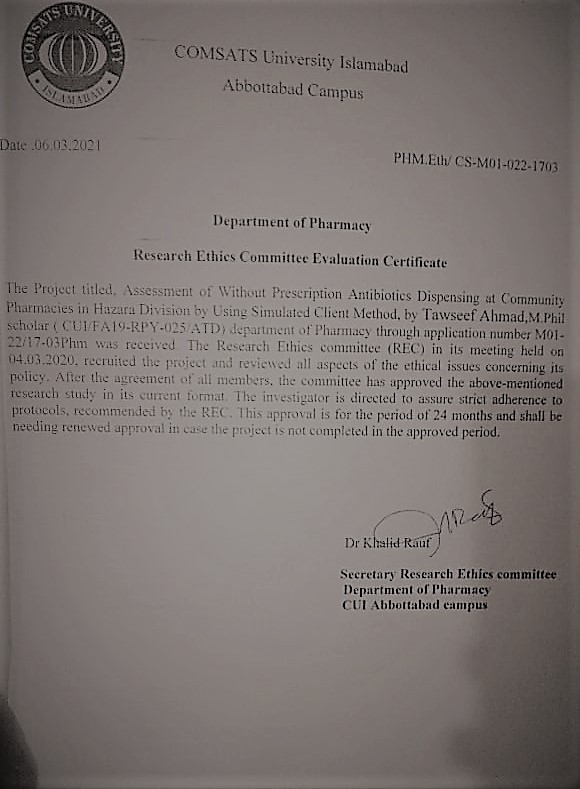

Supplement: S3 File — The approval was taken from Department of Pharmacy COMSATS University Islamabad-Abbottabad Campus. (DOCX) [file pone.0263756.s004.docx]
