# Supplementary material for: Assessment of without prescription antibiotic dispensing at community pharmacies in Hazara Division, Pakistan: A simulated client’s study
Source: PLoS One. 2022 Feb 17;17(2):e0263756. doi: 10.1371/journal.pone.0263756 (PMC8853528; doi:10.1371/journal.pone.0263756)
Supplement: S1 Table — Community pharmacist and dispenser dispensed different dose and strength of antibiotics, the most and least both are shown in Table 5. (DOCX) [file pone.0263756.s005.docx]

**Supplementary file 4: Table 05**: The strength and dose of dispensed antibiotics in Upper respiratory tract infection and Urinary tract infection.

Table 05: Strength and dose of dispensed antibiotics for Urinary Tract Infection

| **Strength+ Dose of dispensing antibiotics** | **Frequency dispensed by community pharmacies/ drug outlets.**  **n (%)** | **Dispensed for a duration to be used (Days)** | **Frequency of pharmacies dispensed antibiotics for days.**  **n (%)** | |
| --- | --- | --- | --- | --- |
| 250 mg BD | 87 (28.1) | 3 Days | 90 (29.0) | |
| 500 mg BD | 85 (27.4) | 5 Days | 178 (57.4) | |
| 400 mg BD | 91 (29.4) | 6 Days | 2 (0.6) | |
| 320 mg BD | 6 (1.9) | 7 Days | 3 (1.0) | |
| 625 mg BD | 4 (1.3) | N/D* | 37 (11.9) | |
| N/D* | 37 (11.9) |  | |  |
| **Total** | **310 (100.0)** | **Total** | | **310 (100.0)** |

*N/D* = not dispensed*

**Strength and dose of dispensed antibiotics for Respiratory tract infection**

| **Strength and Dose of dispensing antibiotics** | **Frequency dispensed by community pharmacies/ drug outlets.**  **n (%)** | **Dispensed for a duration to be used (Days)** | **Frequency of pharmacies dispensed antibiotics for days.**  **n (%)** |
| --- | --- | --- | --- |
| 100 mg OD | 2 (0.6) | 3 Days | 149(48.1) |
| 100 mg BD | 14 (4.5) | 5 Days | 99(31.9) |
| 250 mg OD | 2 (0.6) | 6 Days | 27(8.7) |
| 250 mg BD | 140 (45.2) | 7 Days | 9 (2.9) |
| 500 mg OD | 6 (1.9) | 10 Days | 3(1.0) |
| 500 mg BD | 37(11.9) | 14 Days | 1(0.3) |
| 625 mg OD | 12 (3.9) | N/D* | 22(7.1) |
| 625 mg BD | 44 (14.2) | **Total** | **310 (100)** |
| 400 mg BD | 28 (9.0) |  | |
| 400 mg OD | 3 (1.0) |  |  |
| N/D* | 22 (7.1) |  |  |
| **Total** | **310** |  |  |
